# Noise Producing Toys and the Efficacy of Product Standard Criteria to Protect Health and Education Outcomes

**DOI:** 10.3390/ijerph110100047

**Published:** 2013-12-19

**Authors:** Stuart J. McLaren, Wyatt H. Page, Lou Parker, Martin Rushton

**Affiliations:** 1 College of Health, Massey University, P.O. Box 756, Wellington 6140, New Zealand; E-Mail: w.h.page@massey.ac.nz; 2 Measurement and Product Safety Service, Consumer Affairs, Ministry of Business, Innovation and Employment, P.O. Box 1473, Wellington 6140, New Zealand; E-Mails: lou.parker@mbie.govt.nz (L.P.); martin.rushton@mbie.govt.nz (M.R.)

**Keywords:** noise, toy safety, autism, ISO standards, consumer products

## Abstract

An evaluation of 28 commercially available toys imported into New Zealand revealed that 21% of these toys do not meet the acoustic criteria in the ISO standard, ISO 8124-1:2009 Safety of Toys, adopted by Australia and New Zealand as AS/NZS ISO 8124.1:2010. While overall the 2010 standard provided a greater level of protection than the earlier 2002 standard, there was one high risk toy category where the 2002 standard provided greater protection. A secondary set of toys from the personal collections of children known to display atypical methods of play with toys, such as those with autism spectrum disorders (ASD), was part of the evaluation. Only one of these toys cleanly passed the 2010 standard, with the remainder failing or showing a marginal-pass. As there is no tolerance level stated in the standards to account for interpretation of data and experimental error, a value of +2 dB was used. The findings of the study indicate that the current standard is inadequate in providing protection against excessive noise exposure. Amendments to the criteria have been recommended that apply to the recently adopted 2013 standard. These include the integration of the new approaches published in the recently amended European standard (EN 71) on safety of toys.

## 1. Introduction

For many years, noise in the environment and the workplace has been regarded as a major health and safety issue. It is widely accepted that damage to hearing has an impact on social and economic factors. For children, hearing damage adversely impacts the development of speech and social skills, leading to learning problems. The severity of the impact depends on the type of hearing loss, the level of hearing loss, when it occurred and what interventions is received. There are a wide range of causes of hearing loss in children but noise-induced hearing loss (NIHL) is entirely preventable and should be given due consideration by caregivers and regulatory authorities. There is no recent data available on the incidence of NIHL in young children (6–12 years of age) but the most current data for young American adolescents (12–19 years of age) estimates that 16% [1] have sustained NIHL as indicated by Noise-induced Threshold Shift (NITS), a commonly used marker for noise exposure.

### 1.1. Application of International Workplace Criteria

New Zealand, along with many other countries, has adopted an international criteria for people at work which prescribes an A-frequency weighted, time-average noise level, not exceeding 85 dB over an 8-h period (L_p__Aeq,8h_ ≤ 85 dB) or the equivalent noise energy. This noise exposure can also be expressed as one Pascal-squared-hour or 100% dose. In addition, the criteria prescribe a C or Z weighted peak level of no more than 140 dB (L_p__Cpeak_ ≤ 140 dB). Reid *et al*. [2] state that this limit of 100% dose is based on a trade-off between practicality and protection. There is now a question of whether the maximum 100% dose is applicable in all situations due to the great variation in the influencing factors such as individual susceptibility to NIHL. In 1997 Prince *et al*. [3] re-examined the risk estimates for several different definitions of hearing handicap. Their findings indicate that 4%–8% of the population are expected to suffer noise-induced hearing loss over a 40-year working life being exposed at the 100% dose level.

While the levels of noise-producing toys were reported as early as 1973 [4], the issue of noise exposure in young children has become of increasing concern in the last decade. Yaremchuk *et al*. [5] in 1997 reported on noise levels from commercially available toys in the United States. A difficulty with this paper was an error in reporting peak levels as A-frequency weighted sound pressure levels (SPL) which is clearly incorrect. We have been unable to determine whether the reported data was C or Z frequency weighted peak levels (L_p__Cpeak_), A-frequency weighted maximum SPL (L_p__AFmax_) or time-average levels (L_p__Aeq_). As there are no criteria established specifically for noise exposure to children either at home or in preschools, it has been a common practice to take the international criteria for workplace noise and apply these with some adjustments for young children. Yaremchuk *et al*. [5] applied the Occupational Safety and Health Administration (OSHA) criteria that were current at the time and concluded that exposure to A-frequency weighted levels above 115 dB cannot be considered safe for any duration. This was based on the equivalent energy to the OSHA criteria of an A-frequency weighted time-average level of 90 dB over an 8 h working day (L_p__Aeq,8h_ ≤ 90 dB) with a 5 dB exchange rate as time of exposure was doubled. The international criteria now adopted by many countries are more stringent than the OSHA recommendation used by these authors. This is an A-frequency weighted time-average level of 85 dB for 8 h (L_p__Aeq,8h_ = 85 dB) or equivalent, with a 3 dB exchange rate for the doubling of time exposure used to assess noise exposure in the workplace. Applying this, the equivalent energy to an A-frequency weighted time average level of 85 dB for 8 h is 100 dB for 15 min. It would appear that this rationale was used to determine the Canadian SPL limit of 100 dB as reported by Leroux and Laroche [6]. In 2004, Charonneau and Goldschmidt [7] published a comprehensive assessment of the safety of noisy toys in Canada applying the law of the time. They focused on battery-operated toys designed for the age group 0–3 years. They concluded that although the majority of the toys tested (95%) conformed to the law, 13% did not meet the World Health Organization (WHO) guidelines.

### 1.2. Noise Exposure and Children

The ear canals of the new-born infant are not fully formed at birth and change considerably over the first 24 months of life with changes continuing until around three years of age. A study of the acoustics of the ear canal by Keefe *et al*. [8] reported that it is not until three years of age that the ear-canal development of a child resembles that of an adult. Picard and Bradley [9] indicate that a child’s auditory system does not fully mature until 10–12 years of age. Until maturation, children need a relatively quiet environment to process and understand all the information conveyed to them. This is supported by a number of authors who state that pre-teenage children are immature listeners and therefore need a good acoustical environment for full understanding [10,11,12].

The WHO refers to animal studies which suggest that the hearing of young children is likely to be more sensitive than that of typical adults [13]. The WHO has also stated that no hearing impairment is expected to occur for an A-frequency weighted time-average occupational exposure of 75 dB for 8 h (L_Aeq,8h_). They further state that environmental noise exposure of 70 dB for any duration (L_Aeq,24h_) is unlikely to cause hearing loss in the majority of any sector of the population, even after a lifetime of exposure. This criterion is also supported by Leroux and Laroche [6] who argued that the Canadian regulatory sound limit at that time of 100 dB was excessive. This is a reasonable argument as an A-frequency weighted time-average level for the 15 min exposure is based entirely on the exposure of an adult to workplace noise, with no allowances or correction for young children. They proposed an A-frequency weighted time-average SPL of 75 dB regardless of duration, as a safe limit. Picard and Bradley [14] in their dissertation on speech interference in classrooms, state that noise exposure in young children before the age of six years can induce acute cochlear damage for noise levels and durations of exposure that show no effects in mature subjects. This means that very young children are likely to be particularly susceptible to noise exposure. In addition, children are less able to take avoidance measures when exposed to excessive noise and therefore face a greater risk to their aural health. Such assessments present a strong argument that occupational noise criteria applicable to adult workers, is not appropriate for the protection of young children without suitable adjustment.

### 1.3. ISO Standard for Safety of Toys

Product standards are developed in accordance with data and opinions from various sources along with taking into account the results of available research. Testing for physical response to products is most often carried out on adult and animal subjects and the available data with respect to the physical effects of noise, reflects this.

The International Organization of Standardization (ISO) has developed international criteria for the safety of toys. Part 1 (Safety aspects related to mechanical and physical properties) of ISO 8124 provides criteria on emission levels from sound-producing toys [15]. A number of countries including Australia, New Zealand, China, Malaysia, Singapore and South Africa have largely adopted this toy standard in its entirety. This standard has been revised three times since 2000 to keep up with the rapid development of new toys and manufacturing processes. The International Council of Toy Industries has prepared comparative information of the three major world standards for toys. These are: the International Organization of Standardization ISO 8124-1; the European Union standard EN 71-1; and the American Society of Testing and Materials ASTM F963 standard. With regard to the acoustic criteria of sound-producing toys, EN 71-1 and ASTM F963 have been largely based on ISO 8124-1 criteria with only slight differences [16]. Since the publication of the EU (European Union) Toy Safety Directive (TSD) 2009/48/EC [17] (the general provisions of which took effect in the European market in July 2011, with the chemical requirements coming into force in July 2013) the European Committee for Standardisation (Comité Européen de Normalisation—CEN) has been very active in the development and harmonisation of the EN 71 standard series. The draft amendment to the 2011 version of the EN 71 on acoustics (EN 71-1:2011/prA2:2012 (EN) Safety of toys—Part 1: Mechanical and physical properties—Amendment 2: Acoustics), was recently published as EN 71-1:2011+A2:2013 [18].

Mandatory legal provisions in New Zealand for toys used by children up to 36 months of age are limited to small parts (choking hazards) and lead content (from ingestion) and at present, are based on the 2002 version of the New Zealand standard, AS/NZS ISO 8124.1:2002, Safety of Toys, which is a direct adoption of ISO 8124-1:2000.

The standard in Australia and New Zealand used as a comparator in this research is AS/NZS ISO 8124.1:2010, Safety of Toys [19], which is a direct adoption of the criteria of ISO 8124-1:2009 [15]. The sections of the standard relating to noise are voluntary and offer guidance only. A new version of the standard has been approved this year, 2013 [20], and it is a direct adoption of ISO 8124-1:2012. The new version is essentially the same as the slightly older 2010 standard with respect to noise, except for two small differences. The “Acoustic requirements” sections are identical but in the “Determination of sound pressure levels” section, the 2010 version has the “pass-by” test clause in the “Measurement of continuous sounds” section and in the 2013 version, the equivalent clause is in the “Measurement of impulsive sounds” section. The “pass-by” test is only applied to propelled toys such as hand-actuated spring-propelled toys or other toys that only make a sound when moving. Why this change was made is unclear as one would have expected the pass-by test to apply to both continuous and impulsive sounds when these two sections were created from the “General” measurements clause in appendix F of the earlier 2002 standard. The just published amendment to EN 71 on acoustics [18] takes the sensible approach of including both continuous and impulsive sound measurement for the pass-by tests. The following is a list of toys specifically exempt from the 2002 and 2010 (and 2013) version of the standard:

- Mouth actuated toys (whistles toy trumpets, flutes and so forth)
- Child-actuated toys by muscular action (bells drums squeeze toys, xylophones).

The number of categories of toys and some SPL criteria in the 2010 (and 2013) standard have been reduced from the earlier 2002 [21] standard and the A-frequency weighted SPL descriptor (L_pA_) has been changed to A-frequency weighted equivalent SPL (L_pAeq_). Table 1 shows the toy categories and criteria of the 2002 standard and Table 2 for the 2010 (and 2013) standard.

**Table 1:** Toy categories and criteria of the AS/NZS ISO 8124.1:2002 standard.

Category	Description	Criteria and Sound Descriptors	
Close-to-the-ear toys	A toy designed to emit sound and intended to be used close to the ear (for example toy cellphones)	A-weighted equivalent SPL not exceeding 80 dB	L_p__A_ ≤ 80 dB
		A-weighted emission SPL not exceeding 90 dB when using an ear coupler device for measurement	L_pA_ ≤ 90 dB
		C-weighted peak emission SPL not exceeding 125 dB	L_pCPeak_ ≤ 125 dB
Hand-held toy	Toys designed to emit sound and intended to be held in the hand. Examples given include musical toys, cap firing toys but excludes close to the ear toys	C-weighted peak level not exceeding 125 dB or less	L_p__Cpeak_ ≤ 125 dB
Rattles and squeeze toys	A toy designed to emit sound when shaken or when squeezed by forcing air through an opening. It is intended for children too young to sit up unaided and can be activated by the child or other person	A-weighted single event emission (1 s duration) not exceeding 85 dB	L_p__A,1s_ ≤ 85 dB
		C-weighted peak emission SPL not exceeding 110 dB	L_pCpeak_ ≤ 110 dB
Table-top and floor toys	A toy intended for use on a table top or floor, such as toy cars	C-weighted peak emission SPL not exceeding 125 dB	L_p__Cpeak_ ≤ 125 dB
Cap firing toy	Any toys such as cap guns which use powder/percussion caps	C-weighted peak emission SPL not exceeding 125 dB	L_p__Cpeak_ ≤ 125 dB
All other toys	A toy not fitting the above criteria	C-weighted peak emission SPL not exceeding 125 dB	L_p__Cpeak_ ≤ 125 dB

Note: For all the categories other than the Close-to-the-ear toys, there were no prescribed A-frequency weighted emission sound pressure levels (L_p__A_ dB).

**Table 2:** Toy categories and criteria of the AS/NZS ISO 8124.1:2010 (and 2013) standard.

Category	Description	Criteria and Sound Descriptors	
Close-to-the-ear toys	A toy designed to emit sound and intended to be used close to the ear (for example toy cellphones)	A-weighted equivalent SPL not exceeding 65 dB	L_p__Aeq_ ≤ 65 dB
		C-weighted peak level not exceeding 95 dB	L_p__CPeak_ ≤ 95 dB
Cap firing toy	Any toys such as cap guns which use powder/percussion caps or other explosive action	C-weighted peak level not exceeding 125 dB or less	L_p__Cpeak_ ≤ 125 dB
		No A-frequency weighted equivalent SPL prescribed	
All other toys	A toy not fitting the above criteria	A-weighted equivalent SPL not exceeding 85 dB	L_p__Aeq_ ≤ 85 dB
		A-weighted maximum SPL of 85 dB is used for pass-by tests	L_pAmax_ ≤ 85 dB
		C-weighted peak level not exceeding 115 dB	L_p__Cpeak_ ≤ 115 dB

All three versions of the standard exempt “Radio, tape and CD Players and other similar electronic toys”. However, the 2002 standard specifically states “without headphones or earphones” while the 2010 and 2013 versions has a specific exemption for “Sound emitted from earphones/headphones”. The 2010 standard included a new exemption which has been carried over into the 2013 version: “Toys interfaced-/connected to external devices such as televisions, computers and so forth where the external device controls the sound level”.

The just published amendment to EN 71 on acoustics [18] departs significantly from the previous approach used in this standard that has previously followed the ISO 8124 standard closely. It replaces the existing definitions with the new ones; child actuated toy, mouth actuated toy designed to emit sound, pull-along or push toy, voice actuated toy and self-propelled toy. These new definitions clearly acknowledge how the toys are operated is important. This draft amendment introduced new categories and significantly lowers the highest permissible sound pressure levels compared to the previous standard. The levels are generally 5 dB lower than the equivalent ones in the 2010/2013 ISO standards and thus offer a higher level of protection. The reference for setting the maximum levels is based on the lower action values for noise in the work environment (EU directive 2003/10(EC) [22]) that is L_pAeq,8h_ of 80 dB and L_pCpeak_ of 135 dB. Unfortunately the draft states that “As yet there exists no scientific evidence that the sensitivity of children to loud noise is significantly different from that of adults.” The authors of this paper dispute this statement, as there is ample evidence that young (six years of age or less) children’s hearing is more easily damaged by sound levels that have no effect on adults [13,14]. Eight different toy types have been defined in the draft amendment. They include a new category called “Toys easily confused with Close-to-the-ear toys”. This is essentially toys that by their look and feel are likely to be bought close to the ear even though that may not have been the manufacture’s intention. New toy categories 1, 2 and 3 relate directly to the expected maximum operating time of a toy, termed the “efficient daily operating time”, and correspond to 120 min, less than 40 min and less than 12 min, respectively. The highest permissible SPL increases by 5 dB moving between these categories, corresponding to a constant maximum energy exposure. These new categories directly acknowledge that the expected duration of play is important in setting the highest permissible sound pressure levels.

### 1.4. Typical and Aberrant Methods of Toy Use and Play

Toys are manufactured with typical play patterns in mind. The sound level limits imposed by standards are based on the expected or usual method of play. However, consideration of alternative play methods and reasonably foreseeable abuse must also be made. For example, surface coatings on materials should be free from toxic materials even if it was never intended that toys would be placed in the mouth through the normal course of play. When assessing noise-producing toys, consideration should be made of both typical and atypical or aberrant play methods and the potential for these toys to be used by children younger than the target age. Jenvey and Jenvey [23] in a comprehensive analysis of the importance of pretend play in the development of young pre-school children, show that pretend play requires effective language communication and problem solving skills. Pretend play allows children to practice and develop skills and to explore outcomes. Pretense leads to development of language, communication and problem solving skills. Toys are an important tool in the facilitation of pretend play. They also suggest that children experiencing an intellectual impairment touch and play with toys differently from their typical peers. Children with serious physical and/or intellectual impairments such as the autism spectrum of disorders (ASD), may play and use toys differently to the manufacturer’s intended use. Impairment to imagination is one component of the triad of deficits in ASD which manifests in the lack of pretend play, social deficits and language development problems, with children experiencing this disorder. Play patterns are often characterized by solitary play, which is lacking in imagination and falling below the appropriate level of development [24,25]. These children are more likely to play with objects rather than another person and their obsessive preferences often result in repetitive play with the toy(s) they desire. Young autistic children often demonstrate unusual responses to sounds, some of which they ignore (for example speech), while some sounds they find fascinating and others intensely distressing [24]. Some autistic children can demonstrate an innate ability in music, having perfect pitch and learning music with relative ease. For those with this ability, the interactive type toys such as push button light and musical toys can be very attractive. Such children may be more likely to use a musical or noise producing toy repetitively, or to hold it closer to the ear. In addition to autistic children, others with serious physical and/or intellectual disabilities may find comfort in the interactive type toys which respond with flashing lights or produce a sound at the push of a button.

The development of appropriate play-skills with autistic children is recognized as offering substantial benefits in their childhood development and the means to gain social, cultural and emotional experiences. Additionally, it can result in decreases of inappropriate behavioral problems such as self-stimulatory behavior and tantrums [25]. The use of toys to develop appropriate play is an important part of early intervention strategies with autistic children and Shields [24] has detailed effective routines which can be implemented by parents and caregivers. Sautter *et al*. [25] has also investigated types of toys that can be effectively used to encourage play and interaction between autistic children and their playmates or siblings. Specialists such as speech pathologists often use toys in their clinical practice with children. One parent reported, by personal communication to the authors, witnessing a therapist placing a musical toy right up to the ear of a child to stimulate the child and did not realize the high volume of the sound when placed close to the child’s ear.

The studies examined [23,24,25] all indicate the importance of toys as a valuable aid in the development of typical children and children with impairments such as ASD, although sound-producing toys are not specifically identified. Given the attraction of sound producing toys to children with intellectual impairment, there is now a need for research to identify how such toys can be used to develop educational and development outcomes. It is necessary to also propose appropriate sound level criteria which will not be detrimental to these outcomes or the health and wellbeing of these children. Joubert and Ellis [26] have recommended that future research needs to focus on the length of time that children play with toys as well as the distance between the child’s ears. This statement strongly supports the need for research with children known to engage in solitary and repetitive play for extended periods, such as those experiencing autistic spectrum disorders.

A comprehensive assessment of the safety of noisy toys in Canada [7] in 2004 indicated that children, especially the very young, play with toys in unpredictable or unintended ways. For noise producing toys this means that young children often bring them close to the ears and mouth, even if this is not the expected or intended method of play. They go on to comment that the testing regimes in the standards used precisely prescribed distances at which the sound levels from toys are measured in the laboratory, but according to these authors, such methods do not account for actual use or methods of play. These authors conducted two field trials on play methods and use with a group of young children and found that 22% of the toys evaluated were brought up to the ear (much closer to the ear that the testing distances used in the laboratory testing regimes) and 56% of the toys were used in an unpredictable or unintended manner. Because of this finding they concluded that the majority of toys tested are likely to cause hearing loss over time even with short periods of use (<10 min per day). This strongly supports the argument that such toys should be tested as if they were a Close-to-the-ear toy.

### 1.5. Regulatory Regime in New Zealand

In New Zealand, the Measurement and Product Safety Service, within the Ministry of Business, Innovation and Enterprise (MBIE), is the regulatory authority for the safety of general consumer products. The Ministry has an active educational role in raising the public awareness around safety of consumer products and has taken a particular interest in the safety of toys, including those which produce noise. There are currently no mandatory SPL limits for noise producing toys in New Zealand, although they are covered by the general legislative provisions applying to the safety of any consumer goods.

Products deemed to be harmful or potentially dangerous are covered by the Consumer Guarantees Act 1993, which requires that all goods purchased in New Zealand must be fit-for-purpose, and safe. Part 3 of The Fair Trading Act 1986 enables the Minister of Consumer Affairs to impose specific Product Safety Standards or an “Unsafe Goods Notice” where products are considered to be a safety hazard, and a compulsory product recall can be initiated under the provisions of the Act.

### 1.6. Toys Promoted for Educational Development

In addition to toys manufactured and distributed for general consumption, there are a range of toys manufactured and promoted for special purposes. The most significant toys in this category are those manufactured and promoted for educational development and use. These toys are often premium priced toys as there is an inherent belief that significant research and development has been undertaken to design and manufacture the toys to promote or enhance educational outcomes. The current ISO and Australia/New Zealand standards provide no criteria for such special categories of toys.

Noise levels from such toys are particularly relevant if this hinders or interferes with oral communication during interactive and shared play. If a toy is manufactured and promoted as an educational tool or device that enhances child development and educational outcomes, then it should be fit for that purpose. If a toy discourages speech between children engaged in play or causes children to shout above the noise to be heard (known as the Lombard effect [27]), it generally cannot be deemed to be fit for the purpose of enhancing education, speech or communication outcomes. It is therefore desirable to have appropriate sound level criteria for all such noise producing toys which are manufactured and marketed as educational tools or aids.

### 1.7. Research Aim and Research Questions

The overall aim of this research project was to determine whether or not the changes to the acoustic requirements between the 2002 and 2010 versions of the AS/NZS ISO 8124 standard for safety of toys (Part 1: Safety aspects relating to mechanical and physical properties), has improved the level of health protection. The following specific research questions were proposed to be addressed:

1. What is the level of compliance with the 2010 Australian and New Zealand ISO standard and the earlier 2002 standard for noise-producing toys among the toys collected from merchants and those distributed to special needs children?
2. Did the 2010 revision of the standards provide an increased level of protection?
3. Are the various levels stipulated in the AS/NZS 2010 standards sufficient to protect users from potential harm and risk to hearing damage?

## 2. Methodology

### 2.1. Product Acquisition

A selection of readily available toys were purchased from toy merchants, department stores and toy discount stores. In addition, a small section of noise-producing toys that were distributed as gifts at a regional charitable Christmas party for special needs children over the previous few years, were procured from the personal collection of an autistic child.

### 2.2. Materials

The majority of commercially available toys in New Zealand are imported and sourced internationally from toy merchants and manufacturers. These toys are likely to be representative of the toys available in many countries at the time. In preparation for the Christmas toy buying season, a one day investigation of toys for sale was carried out, covering two large department stores, three discount stores and a well-established toy merchant, all of which were well represented throughout the country.

The noise producing toys were identified in each store and approximately 200 were screened with a hand-held instantaneous reading sound level meter (SLM) to determine approximate sound produced. The toys were removed from their packaging before testing and new batteries where installed. From this initial screening, toys were selected which produced A-frequency weighted SPL in the vicinity of 80 dB or higher at a distance of 25 cm (a measurement distance similar to that used in the standards), or a C-frequency weighted peak SPL of 100 dB or higher at this distance. Twenty-two toys were identified from the initial screening tests as having high readings. These toys were then purchased and sent to the Acoustics Testing Service (ATS) at the University of Auckland, for measurement. ATS is the national center for acoustical testing and is fully ISO accredited.

In addition, a secondary selection of the sound producing toys distributed to special needs children were acquired for evaluation. It wasn’t possible to send these toys to ATS for testing as they were from the personal collections of children and couldn’t be removed from the child for any extended period of time.

### 2.3. Testing Protocol

ATS evaluated the acoustic output of 22 toys purchased commercially using the noise descriptors and test methods specified in the Australian and New Zealand standards AS/NZS ISO 8124.1:2010 and AS/NZS 8124.1:2002. The A-frequency weighted emission SPL (L_pAeq_ dB) for the duration of the test was used for A-frequency weighted emission SPL (L_pA_ dB) in the 2002 standard.

The secondary set of toys consisting of six toys distributed to special needs children, were tested using a Cirrus 831A integrating SLM according to the standards [19,21] in a measurement room compliant with the requirements of the standards. The SLM and its calibrator had current laboratory verification certificates. All care was taken to accurately follow the measurement protocol and methods specified in the standards. 

## 3. Results

The following tables present the results of the testing regime. In accordance with accepted practice, sound pressure levels were rounded to whole numbers. The standards do not prescribe a tolerance to allow for instrument and experimental error. A tolerance of ±2 dB was applied for the assessment, which is a reasonable tolerance for a controlled laboratory situation using a Type 1 SLM. Any level greater than 2 dB above the criterion was deemed a failure. Any level 0–2 dB above the criterion was deemed a marginal pass. Also because both the 2002 and 2010 (and 2013) version of the standard has exemptions for certain types of toys, there was some debate and uncertainty with the following toys over whether these exemptions applied:

- A toy electric drum set which is both a child-actuated toy and also has an electronic volume control. This means that the volume can be determined by both the muscular action of the child (strike level) and also by a volume control.
- Toy juke boxes being a similar electronic toy to a radio or CD player.

Some noise producing toys have a “demo tab”. These are plastic pull tabs that are fixed to the toy’s speaker or battery. These tabs make the toy louder so they can be more easily heard in a busy shop. The tab should be removed before giving the toy to a child.

Table 3 shows the results of the noise assessment and compliance on 22 toys according to the two standards. Going down the table each toy appears in the relevant toy category specified in each standard, along with the criterion applied to determine the level of compliance. Three toys have two entries in the table as they were tested under two different conditions. Four toys clearly failed the 2010 criterion (Reference: #5, #8, #18, #20) and another five toys showed a marginal pass (Reference: #3, #10, #15, #19, #21). Both toys with demo tabs in place (Reference: #2 and #4) failed both standards but passed or marginally passed with the tabs removed for normal usage (Reference: #1 and #3).

**Table 3:** Results of testing to the AS/NZS ISO 8124.1:2010 standard and the earlier 2002 standard.

Toy Description (Name) and Use Category			Sound Descriptors		Compliance	
			L_pAeq_ (dB)	L_pCpeak_ (dB)	2002	2010 ^!^
Ref.	Close-to-the-ear Toys—Criterion	2010	≤65	≤95		
	Close-to-the-ear Toys—Criterion	2002	≤80	None		
#1	Fifi Fun Phone		56	76	Pass	Pass
#2	Fifi Fun Phone (with demo tab in place) ^+^		90	108	Fail	Fail
#3	Fisher Price Learning Phone (with demo tab removed)		66	85	Pass	Marginal Pass
#4	Fisher Price Learning Phone (with demo tab in place) ^+^		89	109	Fail	Fail
#5	Talking Gadget Belt *		91	109	Fail	Fail
Ref.	Hand-Held Toys—Criterion	2002	None	≤125		
	Other Toys—Criterion	2010	≤85	≤115		
#6	Power Gear Gun		70	87	Pass	Pass
#7	High School Musical Microphone		78	92	Pass	Pass
#8	Football Rattle ^#^		92	112	Marginal Pass	Fail
#9	Super Mini Dynamo-Electric Guitar		74	87	Pass	Pass
#10	Project Super Gun		86	102	Pass	Marginal Pass
#11	Silly Sounds Giggle Remote		65	85	Pass	Pass
#12	Black and Decker Chainsaw		78	93	Pass	Pass
#13	Benign Girl Move the Telephone		65	81	Pass	Pass
#14	Rocking Rhythm Electric Guitar		79	94	Pass	Pass
#15	Talking Gadget Belt *		66	84	Pass	Marginal Pass
#16	High School Musical hair brush		70	84	Pass	Pass
#17	Handgun		76	91	Pass	Pass
Ref.	Table-top/Floor Toys—Criterion	2002	None	≤125		
	Other Toys—Criterion	2010	≤85	≤115		
#18	Learning Mower		93	114	Pass	Fail
#19	Cash Register		66	90	Pass	Marginal Pass
#20	Playskool Poundin’ Bedbugs (with hammer)		95	129	Fail	Fail
#21	Playskool Poundin’ Bedbugs (with finger)		80	113	Pass	Marginal Pass
#22	Shak’n Go chicken		76	90	Pass	Pass
Ref.	Rattles—Criterion	2002	L_pAeq,1s_ (dB) ≤ 85	L_p__Cpeak_ (dB) ≤ 110		
	Other Toys—Criterion	2010	≤85	≤115		
#23	Cage Bell (rattle)		81	99	Pass	Pass
#24	TOLO Gripping Activity (rattle)		76	90	Pass	Pass
Ref.	Squeeze Toys—Criterion	2002	≤85	≤110		
	Other Toys—Criterion	2010	≤85	≤115		
#25	Squeeze Rubber Whales		83	98	Marginal Pass	Pass

**^!^** Identical compliance under the 2013 version of the standard; ^+^ Two toys in the “Close-to-the-ear” category did pass the criterion with the demo tabs in place but when the demo tab was removed so that the toys were used as intended, they complied with the 2002 criterion and marginally with the 2010 criterion; * The Talking Gadget Belt was tested in both the hand held toy and close to the ear toy categories as it could fit either category for the 2002 standard; ^#^ The football rattle is not intended as a rattle for very young babies so was not included in the “Rattles” category under the 2002 standard.

### 3.1. Toys Distributed to Special Needs Children

The selection of noise producing toys distributed to special needs children consisted of four jukebox/keyboard toys and a Singing Santa. They were tested according to both the AS/NZS ISO 2010 standard and the earlier 2002 standard and the results are shown in Table 4. Three of the jukeboxes had volume increase/decrease push buttons and each time these devices were switched off and on, they defaulted to the highest (or near) volume setting. This means that a parent or carer has to manually adjust the volume downwards each time the device is switched on, or leave it at full volume. The fourth jukebox and the Singing Santa had no means to control the volume (NV). The Singing Santa also had no method of stopping the song sequence once activated. For this reason, toys were tested at the default volume (DV) once turned on and for those which had volume controls tested again at full volume (FV).

**Table 4:** Compliance of “Other Toys” against the AS/NZ ISO 8124.1:2002 and 2010 standards.

Toy Description (Name) and Use Category			Sound Descriptors		Compliance	
			L_p__Aeq,1min_ (dB)	L_pC__peak_ (dB)	2002	2010
**Ref.**	Other Toys—Criterion	2002	None	≤125		
	Other Toys—Criterion	2010	<85	≤115		
#26	Light Mixer (DV)		86	105	Pass	Marginal Pass
	Light Mixer (FV)		87	105	Pass	Marginal Pass
#27	Protech keyboard (DV)		84	100	Pass	Marginal Pass
	Protech keyboard (FV)		86	102	Pass	Marginal Pass
#28	Mobile DJ Mixer (DV)		95	109	Pass	Fail
	Mobile DJ Mixer (FV)		95	108	Pass	Fail
#29	Talk’n learn Alphabet (NV)		85	109	Pass	Marginal Pass
#30	Lumi-drum (DV)		79	104	Pass	Pass
#31	Singing Santa (NV)		92	107	Pass	Fail

DV (Default Volume when turned on); FV (Full Volume); NV (No Volume control—fixed volume, cannot be adjusted).

In the case of the jukeboxes (Reference: #26–28 in Table 4 above), all defaulted to the highest volume (or near) when they were switched on. The remaining jukebox (Reference: #29) had no volume control. While the Lumi-drum (#30) set was included, there was a question as to whether this standard applies to such musical instrument toys as this toy is strike sensitive. The toys that clearly failed were the Mobile DJ mixer (#28) and Singing Santa (#31).

Table 5 shows a compliance comparison between the 2002 and 2010 versions of the standard for the toys that failed or showed a marginal pass in one or both standards.

**Table 5:** Comparison of compliance between the AS/NZS ISO 8124.1:2002 and 2010 standards for toys that failed or marginally passed.

Ref.	Description	Category		Compliance	
		AS/NZS ISO 2002	AS/NZS ISO 2010	AS/NZS IS0 2002	AS/NZS IS0 2010
#5	Talking Gadget Belt	Close-to-the-ear	Close-to-the-ear	Fail (L_Cpeak_)	Fail (L_PAeq_ & L_Cpeak_)
#8	Football rattle	Hand-held	Other	Pass	Fail (L_PAeq_)
#18	Learning Mower	Table-top/floor	Other	Pass	Fail (L_PAeq_)
#21	Playschool Poundin’ Bedbugs (with hammer)	Table-top/floor	Other	Fail (L_Cpeak_)	Fail (L_PAeq_ & L_Cpeak_)
#25	Squeeze Rubber Whales	Rattle/Squeeze Toy	Other	Marginal Pass	Pass
#28	Mobile DJ Mixer	Table-top/floor	Other	Pass	Fail (L_PAeq_)
#31	Singing Santa	Hand-held	Other	Pass (L_PAeq_)	Fail (L_PAeq_)

## 4. Discussion

The first research question examined compliance of a selection of toys with both the 2010 standard and the earlier 2002 version. A total of 28 toys were tested, with six toys in the test failing to meet the 2010 standard (21% failure). Two of the toys, the Talking Gadget Belt (#5) and the Playskool Pounding Bedbugs actioned with hammer (#20), failed both the 2010 and 2002 standards (7% failure). It is worth noting that these two toys failed on both equivalent sound level (L_pAeq_) and peak level (L_pCpeak_) criteria in the 2010 standard, whereas they only failed on peak level criteria in the earlier (2002) standard. Four toys, the football rattle (#8), learning mower (#18), Mobile DJ mixer (#28) and Singing Santa (#31), failed the 2010 standard criteria but complied with the earlier 2002 standard criteria. This was due to the introduction of A-frequency weighted equivalent SPL criteria to cover all non-categorized toys in the 2010 standard. Two toys with “demo tabs” (#2 and #4) in place, failed both standards but passed or marginally passed when the tab was removed for normal operation.

The second research question examined if a greater level of protection was provided by the 2010 standard when compared to the earlier 2002 standard. While this work gives a reasonable indication that the 2010 standard provides a higher level of protection for a range of toys, than the earlier 2002 standard, there is one high risk category which appears to be the exception. The Squeeze Rubber Whales (#25) which only marginally passed the 2002 standard by applying a +2 dB tolerance to allow for experimental error, now clearly passes the 2010 standard. This appears to be the one case where the 2010 (and the subsequent 2013) standard do not provide increased protection. The removal of the category for rattle and squeeze toys from the later standards are the primary reason for this difference. The 2010 (and 2013) standards specifically exempt child actuated toys, apart from rattles, which are claimed to be covered under impulse sound level requirements. We believe this statement to be a flawed analogy as this assumes that sounds emitted from these toys are restricted to impulse sounds (L_pCpeak_). The toy in question produces a continuous high-pitched sound when actioned. Impulse sound and the continuous equivalent sound level (L_pAeq_) are different descriptors and it must not be assumed that one of these metrics will automatically cover the other. Since these toys are for babies and young infants, we question the rationale of removing the rattle and squeeze toy requirements for the most vulnerable group of children. The 2010 (and 2013) standard may not provide the potential protection from harm as achieved in the earlier 2002 standard for these toys. Furthermore, it appears to be based on the assumption that it is only the baby that is going to actuate these toys when it is highly likely that other children and adults will actuate these toys around babies without clearly realizing the potential danger.

The third research question explored the various sound emission levels prescribed in the 2010 (and 2013) standard and investigated whether these were sufficient to protect users from potential harm and risk to hearing damage. There seems to be a general acceptance that the A-frequency weighted equivalent SPL suggested by the WHO [13] of 70 dB over 24 h (L_Aeq,24h_), would be the ideal level for rattles and squeeze toys and, with some adjustment, for toys held next to the ear. The authors believe the current criteria of L_p__Aeq_ ≤ 85 dB is excessive and has the potential to cause hearing damage if such levels are frequently experienced close-to-the-ear in young children. An A-frequency weighted equivalent SPL of no more than 80 dB should be considered as the criteria (L_pAeq_ ≤ 80 dB), in-line with the value specified in the recently amended EN 71 [18]. In addition, a separate category for rattle and squeeze toys needs to be reinstated and we recommend an equivalent sound pressure level of no more than 75 dB (L_pAeq_ ≤ 75 dB) and an impulse level of no more than 95 dB (L_pCpeak_ ≤ 95 dB). This will provide greater protection for the vulnerable young infants that these toys are targeted at. The criteria for “Close-to-the-Ear” toys in the 2010 and 2013 standards (L_pAeq_ ≤ 65 dB and L_pCpeak_ ≤ 95 dB) significantly increased the level of protection from the earlier standard.

In the case of toys intended to be held in the hand or those used on the floor or table-top, which are now included in the “other” category of toys, there is ample evidence that these toys are not always played in the manner envisaged by manufacturers. The current A-frequency weighted sound levels do not take into account that there is nothing to stop any child putting these toys up to their ear, or placing their ears over the top of speaker outlets. Because a toy is not designed to be placed in the mouth or chewed as part of the normal or intended course of play, it does not mean that this will not happen. An A-frequency weighted equivalent SPL of no more than 80 dB should be considered as the criteria (L_pAeq_ ≤ 80 dB). Rattles and squeeze toys should not emit an A-frequency weighted single sound level of more than 75 dB because although they are designed for very young children it is likely they will be operated by older children or adults.

Excessive impulse sound levels (L_pCpeak_) can also potentially cause serious damage to hearing in young children. The AS/NZS ISO 2010 standard made substantial reductions to 115 dB, except for cap firing toys where the peak level of 125 dB remains. However, this requires a warning of potential hearing damage on any toy which exceeds a peak level of 110 dB. It is therefore essential that impulse (peak) level criteria for the non-categorized (Other) toys should be reduced to 110 dB, except for cap firing toys, if they cannot meet this criterion. In this case, clear warnings of potential harm should be included on the package of the toy. All these recommended values would provide a greatly enhanced level of protection than currently prescribed and should be subject to review, once more information becomes available justifying further amendment. Applying these proposed amendments to the toys that were tested in this work, a further seven toys (25%) would fail. It should be noted that none of the toys tested were self-propelled and so the “pass-by” clauses of the standards were not exercised. However, ambiguity exists in testing such toys in both the 2010 and 2013 standards and this should be resolved in a future revision.

### 4.1. Atypical Methods of Toy Play

In Section 1.4 of the Introduction, the authors considered the potential risk of hearing damage during atypical methods of play with toys. Given that such patterns are often seen with ASD children and usually include extended periods of use or repetitive play, there is a strong possibility that this may result in greater harm for the level of sound produced by the toy. Children will not always play with toys in the way that the manufacturer envisaged through the normal or intended course of play. A toy normally used on a table-top or held in the hand can readily be placed close to the ear either by lifting the toy to the ear or the child placing their ear over the speaker while on the table or floor. This type of play is a typical pattern of behavior with autistic children. The most effective way to address this issue is to reintroduce a category for table-top toys with an A-frequency weighted SPL, similar to that for close-to-the-ear toys and to introduce an expected maximum duration of usage criterion. In the case of those with variable volume, it seems unreasonable to expect a young child or carer to manually adjust the volume every time the unit is switched on. The volume setting when turned off should remain at the same setting when turned on again. This is a common feature of other kinds of audio equipment such as televisions, sound systems, portable media players and so forth. Although there is a foreseeable pattern of use in the way that a toy is operated, the standard also requires testing to be carried out with consideration of reasonably foreseeable abuse. The recently published amendment to EN 71 [18] partially acknowledges this issue with the creation of the new category “Toys easily confused with Close-to-the-ear toys”. Given the knowledge that serious, permanent, but preventable harm, may be caused by excessive exposure to noise, it is a small step to take a precautionary approach and account for atypical patterns of usage in setting criteria in standards.

### 4.2. Toys Promoted for Educational Development

In Section 1.6 of the Introduction, the authors stated that the current ISO and Australia/New Zealand standards provide no specific criteria for special category toys. There is a significant class of special category toys, namely those which are manufactured and promoted as educational tools and/or for enhancing educational outcomes, which may benefit from having specific sound emission criteria. The rational for this is not around decreasing the risk of hearing damage, but ensuring the toys are fit-for-purpose, in particular that they do not significantly interfere with speech and oral communication when used as intended. Most toys are generally used in an environment with other people present, be they similar aged children, or caregivers. Also, the sharing of a toy is to be strongly encouraged in play to enhance social learning, but if the toy is too loud for easy communication without raising the voice, there is a health/learning issue. The SPL of an adult conversation at a normal conversation distance is 60–65 dB and for children this is likely to be significantly lower. A general guideline for effective speech intelligibility is that the signal to noise ratio be 15 dB or higher [28]. This means that if the toy emits sound at 70 dB (L_pAeq_) then a speech signal should ideally be at 85 dB which is well above normal speech levels, especially that of a young child. If a toy emits a level of noise which degrades communication, a vital component of shared play, then there is a fit-for-purpose issue if the toy claims to advance social and education outcomes. Twin boys of 4 years old were observed playing with a toy that is promoted on as enhancing the following outcomes:

- Imagination—encourages the child to enjoy using their imagination
- Social skills (interactive share play aspect). Helps the child learn how to make friends and enjoy company

The boys had to significantly raise their voices to communicate with each other and there was no way of adjusting the sound level. The toy easily complied with the 2010 standard for sound emission “other toys category” but was not considered by the authors to be fit-for-purpose in achieving the social skills with interactive shared play outcome of the manufacturer. This very preliminary finding is an avenue for future study.

### 4.3. Acoustical Parameter Tolerance

While the ISO standards do provide tolerances for the physical parameters such as measurements and application of force, no prescribed acoustical parameter tolerance is provided to allow for instrument or experimental error. This deficiency needs to be addressed to allow for reasonable and practical interpretation of sound level criteria when assessing noise producing toys. The most recent AS/NZ ISO 8124.1:2013 standard (and the 2010 version) allows for two types of acoustic test environments, meeting the qualification requirements specified in Annex A of ISO 3746:2010 *Acoustics—Determination of sound power levels and sound energy levels of noise sources using sound pressure—Survey method*, or the more accurate ISO 11201:2010 *Acoustics—Noise emitted by machinery and equipment—Determination of emission sound pressure levels at a work station and at other specified positions*. This more accurate test environment specifies three grades with associated maximum achievable accuracy. The “Grade 2 (Engineering)” would appear most applicable and practical in this situation, with a specified maximum achievable accuracy ≤2 dB. This is also the recommended test environment in the draft amendment to EN-71 (EN 71-1:2011/prA2:2012). Thus a tolerance of +2 dB from the criteria is reasonable and practical.

### 4.4. Limitations of the Study

A potential limitation of the evaluation of the primary set of toys is the total number of toys tested. Although over 200 noise-producing toys were screened at the toy outlets by a handheld SLM, resulting in the selection of 22 toys, there was not 100% coverage and only major toy supply outlets were included. It is the experience of the authors that noise producing toys sold at low cost outlets are often excessively noisy, even though the total number of toys sold from such outlets as a proportion of the market is small. The other limitation of this study is that the toys tested were limited to those available in New Zealand in 2010. There has been a general trend in recent years by toy manufactures for toys to become more complicated with flashing lights and sounds in an effort to appeal to children raised around television and the internet, so the proportion of toys available internationally that produce noise is likely to have increased since this study was completed.

The evaluation of secondary set of toys, those distributed to special needs children, has more significant limitations. The most obvious limitation is that this toy selection is a specific sample and may not be representative of toys purchased by parents and caregivers of such children. The second limitation is that it was not possible to send these toys away to ATS for the acoustical testing. Although the authors took all care in performing the acoustical measurements consistent with the procedures outlined in the standards, it is likely that these measurements will be less accurate compared to those from ATS for the primary set of toys.

## 5. Conclusions

The level of compliance to the 2010 (and 2013) version of the standard was reasonably high at 79%. Overall the 2010 (and 2013) version of the standard provides greater protection from hearing damage that the earlier 2002 version. However, one toy that passed the 2010 standard failed the 2002 standard due to the removal of a specific toy category. Whether or not the criteria in the 2010 standard provide sufficient level of protection from hearing damage is less clear. But the authors believe the current criteria of L_pAeq_ ≤ 85 dB is excessive and has the potential to cause hearing damage in children if such levels are frequently experienced close-to-the-ear. This assertion is further supported by the new criteria in EN-71 [18] that set the level at L_pAeq_ ≤ 80 dB. Furthermore, there is secondary evidence [9,14] that the criteria in the standards fails to recognize that the hearing of infants and young children may be damaged by much lower levels of noise, for shorter periods of exposure. Also, due to the atypical toy play methods often displayed by children with ASD [23], such as prolonged and repetitive use, there is a strong possibility these children are at greater risk of hearing damage.

## 6. Recommendations

It is therefore recommended that the following amendments are made to the current AS/NZ ISO 8124.1:2013 standard using the existing test methods.

1. Revise the scope of the standard to ensure the inclusion of Rattle and Squeeze Toys with the maximum allowable A-weighted equivalent sound level criteria of 75 dB (L_pAeq_ ≤ 75 dB) and the impulse sound criteria to 95 dB (L_pCpeak_ ≤ 95 dB).
2. Introduce a requirement of permanent volume controls on all jukebox type toys, with the default volume when switched on to be set at the lowest level.
3. Introduce an acoustic parameter tolerance of +2 dB for the prescribed criteria.
4. Reinstate the “pass-by” clause (present in the A/NZS ISO 2010 standard—ISO 2009) in the “Measurement of continuous sounds” section.
5. Consider the integration of the new approaches taken in the recent amendment to EN 71 (EN 71-1:2011+A2:2013) on acoustics, into the relevant clauses of AS/NZS ISO 8124.1.

The level of non-compliance against the proposed amended criteria would increase the number of failures among the toys tested to 12 (42% failures). Based on this estimation a significant number of toys on the market could pose an unacceptable risk to children.

Consideration should also be given to reducing the maximum allowable A-frequency weighted equivalent sound level to 80 dB (L_pAeq_ ≤ 80 dB) and the maximum allowable peak levels to no more than 110 dB (L_pCpeak_ ≤ 100 dB) for non-categorized toys (Other category).

It is anticipated that adoption of these recommendations would have the benefit of significantly reducing the future social and economic cost to Australia, New Zealand and any country which has adopted this ISO standard. It is likely that this can be achieved with minimum compliance costs to business and few regulatory implications. However, since the recommendations apply to sections of the standard that are currently voluntary and offered as guidance only, in both New Zealand and Australia, the benefits will not be realised until there is full compliance by toy importers and local toy manufacturers.
